# Epiretinal Membrane Surgery in Daily Clinical Practice: Results of a Proposed Management Scheme

**DOI:** 10.1155/2019/8246858

**Published:** 2019-01-10

**Authors:** Jesús Pareja, Alba Coronado, Inés Contreras

**Affiliations:** ^1^Clínica Rementería, Madrid, Spain; ^2^Instituto Provincial de Oftalmología-HGU Gregorio Marañón, Madrid, Spain; ^3^Hospital Universitario Ramón y Cajal. Instituto Ramón y Cajal de Investigaciones Sanitarias (IRYCIS), Madrid, Spain

## Abstract

**Purpose:**

To report the results of the epiretinal membrane (ERM) management guidelines followed in our center.

**Methods:**

Patients with ERM seen between 2014 and 2015, with ≥2 years follow-up or who had undergone ERM surgery, were included. Corrected visual acuity (VA), lens status, and ERM configuration were recorded at each visit. Our guidelines for ERM are if VA is ≥20/30, observation is recommended unless there is moderate/intense metamorphopsia. Vitrectomy is recommended during follow-up if there is a drop >one line in VA with changes in ERM configuration. If VA at diagnosis is <20/30, vitrectomy is recommended. If visual loss is thought to be due to cataract, phacoemulsification is performed first and visual status reevaluated.

**Results:**

Ninety-nine eyes of 94 patients were included; 52 eyes underwent vitrectomy, and 47 eyes were monitored. From eyes with VA at diagnosis <20/30 (41 eyes), 8 eyes underwent isolated phacoemulsification: VA improved to ≥20/30. Vitrectomy was recommended but refused by 4 patients. The other 29 eyes underwent vitrectomy. Of the 58 eyes with VA at diagnosis ≥20/30, 5 underwent surgery due to metamorphopsia. Eighteen eyes underwent vitrectomy during follow-up. VA improved a mean of 0.13 logMAR (SD 0.30) after vitrectomy. There were no differences in mean VA improvement between eyes that underwent vitrectomy within six months of diagnosis (0.24, SD 0.32) and those that underwent surgery more than six months after diagnosis (mean 0.17, SD 0.17), *p*=0.106. Three eyes developed postsurgical complications with visual loss: persistent macular edema in one eye, two consecutive retinal detachments in one eye, and a central visual defect in another eye. At the end of follow-up, VA was similar in the observation group (0.14, SD 0.14) and in the vitrectomy group (0.16, SD 0.28), *p*=0.528.

**Conclusions:**

Our proposed guidelines lead to visual preservation in most patients while limiting surgery and its possible complications.

## 1. Introduction

Epiretinal membranes (ERMs) are sheet-like fibrous structures that develop on the surface of the retina. They produce few symptoms, but in some patients, ERM contraction leads to a distortion of the foveal structure, causing visual loss and/or metamorphopsia. Surgical removal of the ERM usually leads to improved visual acuity and decreased metamorphopsia [1]. However, there are no clear guidelines to determine when vitrectomy should be performed, favoring early surgery after diagnosis is the fact that the main prognostic factor is preoperative visual acuity [2]. But although modern vitrectomy is associated with few complications [3], some patients undergoing surgery do experience visual loss and recent studies comparing watchful waiting (with vitrectomy in case of disease progression) with early surgery in patients with good visual acuity suggest that visual improvement is similar in both groups [4].

With the increasing number of elderly people in our society and the better access to ophthalmic care and imaging techniques such as optical coherence tomography (OCT), the number of patients diagnosed with the ERM is expected to be ever greater [5, 6]. It is therefore necessary to search for an optimized treatment strategy, which would reduce unnecessary surgery while ensuring good visual outcomes.

The purpose of this retrospective study is to report the results of ERM management guidelines followed in our center, evaluating the visual outcomes of patients diagnosed with the ERM visited in a two-year period, both those who underwent surgery and those who were followed-up.

## 2. Methods

The electronic database of our center was used to identify all patients seen in a two-year period (2014-2015) with the diagnosis of ERM. The records of all these patients were reviewed. Patients were included in the study if they had at least two years follow-up or if they had undergone surgery for ERM in our center during this period. Patients with coexisting ocular pathologies were not excluded, except for those with advanced pigmentary retinosis. Institutional review board approval was obtained. The study adhered to the tenets of the Declaration of Helsinki.

The data that were recorded for all patients included year of ERM diagnosis, age at diagnosis, gender, and presence of other ocular pathologies. Corrected visual acuity, lens status, ERM configuration, and central subfield thickness in spectral domain OCT were recorded for all patients at diagnosis, as well as six, twelve, and twenty-four months after diagnosis for nonoperated patients and at the last follow-up visit. For those patients who underwent surgery, the same data were recorded just prior to surgery, as well as time between diagnosis and surgery, type of surgery (vitrectomy or combined phacovitrectomy), and development of intra- or postsurgical complications. Corrected visual acuity, lens status, foveal structure, and central subfield thickness were recorded six, twelve, and twenty-four months after surgery, as well as at the last follow-up visit. However, no minimum follow-up was required for patients who had undergone vitrectomy; this decision was taken in order not to miss patients who might have had a negative visual outcome and might have decided to consult elsewhere. There is as yet no consensus on the classification of ERMs: several groups have proposed different classifications [7–9]. In a slight modification from Konidaris et al.'s proposed scheme [8], we classified membranes as preserved foveal pit, absence of foveal pit, prominent retinal thickening, anteroposterior traction, and anteroposterior traction with retinal edema.

The following guidelines are followed in our center for patients with an ERM: if visual acuity is equal or better than 20/30 at diagnosis, observation is recommended unless the patient has moderate or intense metamorphopsia. Patients receive information on the symptoms associated with ERM progression and are advised to consult promptly if they develop them. Patients are initially seen every 3 months; if no change is observed after two visits, patients are followed twice yearly. Surgery is recommended if there is a drop of more than one line in visual acuity with changes in the ERM configuration on OCT which suggest visual loss is due to membrane progression. If visual acuity at diagnosis is lower than 20/30 and visual loss is deemed to be due to the presence of the ERM, vitrectomy is recommended. Both at diagnosis and during follow-up, if visual loss is thought to be due to cataract progression, cataract surgery is performed and visual status is reevaluated one month later. Combined phacovitrectomy is performed if the ERM is deemed to be the main cause of visual loss but there is also significant lens opacity.

In our center, three-port pars plana vitrectomy is performed under retrobulbar anesthesia, in most cases with 25G instruments. Surgeons conduct a nearly complete vitrectomy and indentation to evaluate the retinal periphery. Any tears detected are treated with laser. After posterior hyaloid removal, the ERM and internal limiting membrane (ILM) are stained with dual blue. The ERM is grasped and peeled with end-gripping forceps. If necessary, the ILM is restained to improve visualization prior to removal. If combined phacovitrectomy is scheduled, lens surgery is performed as the first step of surgery. Postoperatively, patients are treated with topical antibiotics for one week and corticosteroids tapered during the first month.

Statistical analysis was performed with the SPSS program (version 20.0, SPSS Inc., Chicago, Illinois). Visual acuity was converted to LogMAR units prior to analysis. For categorical variables, number (*n*) and percentage (%) are presented. For continuous variables, mean, standard deviation (SD), and range are provided. For comparisons between groups, the chi-square exact test was used for categorical variables and the Mann–Whitney *U* test was used for continuous variables. For comparison within groups, the Wilcoxon signed-rank test was used. A *p* value <0.05 was considered statistically significant.

## 3. Results

A total of 272 eyes of patients visited between 2014 and 2015 were identified as having an ERM in our electronic database. Of these, 20 eyes were excluded because they had a macular pseudohole, 11 eyes because they had a lamellar macular hole, and one eye because it had vitreomacular traction. Seventy-six eyes were excluded because they had a very thin ERM with no foveal distortion and 35 eyes because although they had an ERM, they did not have two years follow-up. Five eyes were excluded because they had advanced pigmentary retinosis and 25 because they had undergone ERM surgery elsewhere and no data were available on preoperative visual acuity. Thus, 99 eyes of 94 patients were included in the study: 52 eyes underwent vitrectomy (Group 1) and 47 eyes were followed-up for at least two years (Group 2).

**Figure 1:**
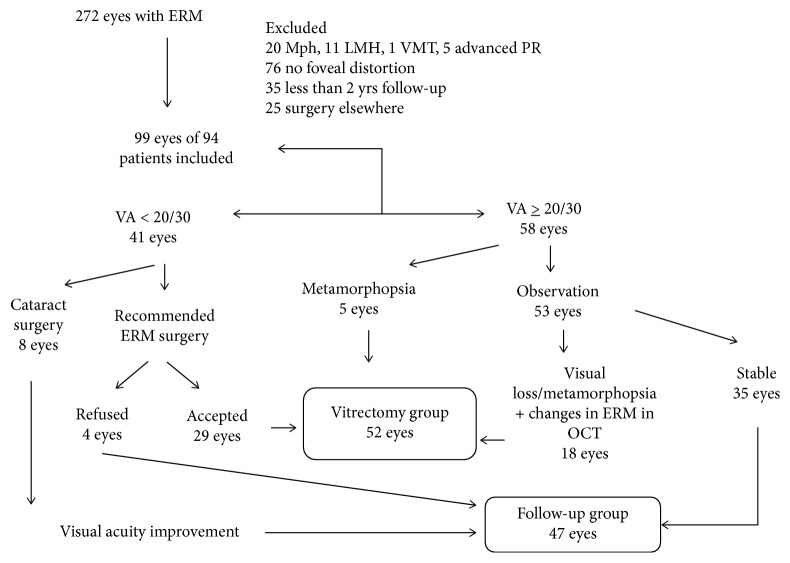
Flowchart showing patients included in the study, in the vitrectomy or follow-up group. ERM, epiretinal membrane; MPH, macular pseudohole; LMH, lamellar macular hole; PR, pigmentary retinosis; yrs, years; OCT, optical coherence tomography.

The age and gender of patients at the time of ERM diagnosis were similar in both groups. Mean age of patients who underwent surgery was 70.5 years (SD 6.7, range 56 to 83 years) with 63.5% of men and that of patients who underwent observation was 71.4 years (SD 7.8, range 58 to 93 years) with 57.4% of men.  Table 1 records the characteristics at diagnosis in both the groups.  Figure 1 shows how the patients were managed depending on initial visual acuity and on visual and ERM changes during follow-up. From the eyes with a visual acuity at diagnosis of less than 20/30 (41 eyes), visual loss was deemed to be due to lens opacification in 8 cases. These eyes underwent cataract surgery: visual acuity improved to 20/30 or better and vitrectomy was not considered necessary. Visual acuity remained stable during follow-up. In 4 other patients, vitrectomy was recommended but the patients refused surgery, since they believed themselves to be too old; their ages ranged between 78 and 93 years. The other 29 eyes underwent vitrectomy; in 3 eyes, surgery was delayed more than six months due to patients' personal problems. Of the eyes with a visual acuity at diagnosis of 20/30 or better, 5 underwent early surgery due to moderate or intense metamorphopsia. The other patients were followed up and underwent surgery if there was a visual loss of more than one Snellen line or increased metamorphopsia with membrane progression on OCT which would explain visual changes. Thus, one eye underwent early surgery (less than 6 months after diagnosis) due to a decrease in visual acuity from 20/20 to 20/50. A further 17 eyes underwent vitrectomy during follow-up.

Isolated vitrectomy was performed in 39 eyes (75%) and combined phacovitrectomy in the remaining 13 eyes (25%). There were no intrasurgical complications. Seven eyes (13.5%) developed postsurgical complications. Four eyes had postsurgical macular edema, which was controlled in two cases with long-term treatment with topical NSAIDs and with periodic intravitreal injection of corticosteroids (dexamethasone implant) in the other two eyes. One eye required rotation of a toric intraocular lens. Two eyes had a retinal detachment which was repaired with a second vitrectomy. One of these eyes developed a redetachment, which required silicone oil tamponade. The ERM recurred in one eye, which underwent a new vitrectomy. During follow-up, one eye developed a central retinal vein occlusion which required intravitreal injections of antivascular endothelial growth factor and dexamethasone implant; this was not clearly related to the initial vitrectomy since it developed more than three months later.

Of those eyes that underwent surgery, 13 eyes (25%) were operated within one month of diagnosis, 26 eyes (50%) within 3 months, and 41 eyes (76.9%) within one year. Mean time between diagnosis and surgery was 10.4 months (SD 15.6 months), with a range from less than one month to 72 months after diagnosis. Visual acuity improved a mean of 0.13 logMAR units (SD 0.30) after surgery. There were no differences in mean visual acuity improvement between eyes that underwent surgery within six month of diagnosis (mean improvement 0.24 logMAR, SD 0.32) and those that underwent surgery more than six months after diagnosis (mean 0.17, SD 0.17), *p*=0.106. Visual acuity improved two or more Snellen lines in 29 eyes (55.8%), remained stable in 16 eyes (30.8%), and worsened by two or more lines in 7 eyes (13.5%). Visual loss was due to postsurgical complications in three eyes: persistent macular edema in one eye (this patient also had a cerebrovascular accident which may have contributed to visual loss), two consecutive retinal detachments in one eye, and the development of a central visual defect in another eye (there was no other explanation for this visual loss). In one eye, a prior atrophy due to age-related macular degeneration advanced, one eye developed proliferative diabetic retinopathy, and one eye developed posterior capsule opacification which was pending treatment at the time of the last follow-up visit. In one eye, visual acuity dropped from 20/16 to 20/20 for no clear reason.

**Figure 2:**
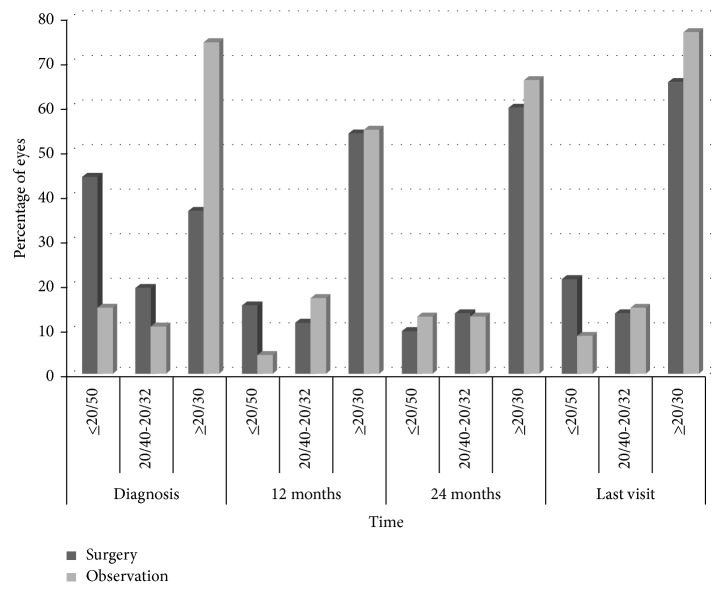
Visual status of eyes with epiretinal membrane in the vitrectomy and observation groups at diagnosis and during follow-up.

In the follow-up group, visual acuity improved by two or more lines in 15 eyes (31.9%); this improvement was due to cataract surgery. Visual acuity remained stable in 26 eyes (55.3%) and worsened in 6 eyes (12.8%). Visual loss was due to ERM progression in two eyes of patients who had refused vitrectomy. It was deemed to be due to cataract progression in three eyes (of patients who did not wish to undergo phacoemulsification since they had no difficulties in their daily activities) and in one phakic eye in which the spontaneous release of the ERM lead to a decrease in visual acuity from 20/20 to 20/30.  Table 2 shows the visual status of both the groups at the last follow-up visit, and Figure 2 describes the distribution of visual acuity in both the groups.

At ERM diagnosis, only 9 eyes (19.1%) of the follow-up group compared to 22 eyes (42.3%) of the surgery group were pseudophakic. One year after vitrectomy, 39 eyes (75%) in the surgery group had undergone cataract surgery; at the last follow-up visit, the number had increased to 49 eyes (94.2%). In the follow-up group, 19 eyes (40.4%) had undergone cataract surgery one year after ERM diagnosis, increasing to 33 eyes (70.2%) at the last follow-up visit.

## 4. Discussion

Recent advances in vitreoretinal surgery have made ERM membrane surgery a very safe procedure [3, 10]. Together with the fact that in almost all reports, preoperative visual acuity is the main prognostic factor of postoperative visual acuity [2, 11, 12], this is leading to surgery being proposed to patients with very good visual acuities. However, we must take into account that complications can occur after pars plana vitrectomy, leading in some cases to irreversible visual loss, and that the number of patients with ERM is expected to increase with the aging of the population [6]. Furthermore, most published reports exclude patients with ocular pathologies other than the ERM and might therefore communicate visual outcomes better than what would be expected in a real clinical practice setting. In this paper, we present the results of our management scheme for patients with ERM, which tries to reach a compromise between preserving good visual acuities and avoiding unnecessary surgery. We excluded only patients with advanced pigmentary retinosis due to the very special characteristics of this disease. We decided not to establish a minimum follow-up period in eyes undergoing surgery because patients with negative visual outcomes might decide to seek a second opinion elsewhere and might be lost to follow-up. We did establish a minimum follow-up period for patients who were followed-up because we wished to evaluate how these patients fared in the long run.

The visual acuity threshold for indicating surgery in patients with the ERM was set at 20/30 because the difficulties for performing instrumental activities for daily living doubles between 20/25 and 20/32 [13]. Since preoperative visual acuity determines postoperative acuity, this would preserve the patient's visual capacity. In our study, surgery leads to a mean improvement in logMAR visual acuity of 0.13 (from a mean of approximately 20/40 to 20/30), with 55.8% of eyes improving at least 2 Snellen lines. These results are similar to those achieved by other groups in eyes with good initial visual acuities: Reilly et al. reported an increase from 0.305 logMAR (20/40) to 0.250 logMAR (20/35) and Lehpamer and Carvounis from 20/40 to 20/28 one year after surgery [14, 15]. We found no statistically significant differences in visual improvement between eyes that underwent early surgery (less than 6 months after diagnosis) and those that underwent delayed surgery, in accordance with the results of Kofod et al. [4]. It seems that, whatever the trigger for ERM formation is, ERM develops and progresses quickly so that by the time most patients consult, the ERM is quiescent. Thus, more than 75% of eyes underwent surgery within one year after diagnosis, suggesting that progression is rare from then on. Previous studies have also shown this stability [16].

In spite of the good visual outcomes achieved in most patients, two eyes did experience visual loss due to postsurgical complications. One eye had two consecutive retinal detachments, which led to a final visual acuity of 20/400 (from an initial of 20/60). Another eye developed a central visual field defect. The development of visual field defects has been reported after uneventful vitrectomy and may be due to difficulties in optic nerve head flow during surgery because of increased intraocular pressure levels [17], or else to nerve fiber layer defects produced by intrasurgical manipulation [18]. In fact, two test points within 10° eccentricity in standard automatic perimetry have been shown to have a sensitivity significantly and reproducibly decreased after vitrectomy in glaucomatous eyes [19]. These cases underline the fact that every surgical procedure has its risks and supports the possibility of observation in eyes with good visual acuity and no signs of ERM progression.

In our follow-up group, visual acuity worsened due to ERM progression only in two eyes of patients who had refused surgery; curiously, ERM resolution led to a decrease in visual acuity in one eye, although this was a phakic eye and cataract progression might have contributed to the visual drop from 20/20 to 20/30. When in doubt as to the contribution of ERM to visual loss in phakic eyes, we performed isolated phacoemulsification. This allowed us to avoid vitrectomy in many cases. Previous studies have shown that this two-step approach does not worsen visual outcomes if vitrectomy is finally necessary [20].

Limitations of our study are those common to all retrospective reports, mainly that a lot more patients were seen with ERM that were not included because they were not followed-up for at least two years or because they had undergone surgery elsewhere. The strength of the study is that the three surgeons in our practice consistently follow the same guidelines.

In summary, our proposed management guidelines for ERM with vitrectomy recommended for patients with metamorphopsia or a visual acuity lower than 20/30 at diagnosis or with visual loss due to progression of ERM on OCT during follow-up lead to visual preservation in most patients while limiting surgery.

## Figures and Tables

**Table 1:** Characteristics at the time of diagnosis of epiretinal membrane. Values provided for visual acuity and central macular thickness are mean (standard deviation) and range.

	Observation group	Vitrectomy group
Number of eyes	47	52

LogMAR visual acuity	0.14 (0.14) Range 0.5 to 0.0	0.33 (0.26) Range 1.30 to −0.10

Central macular thickness (*µ*m)	390 (47) Range 266 to 484	451 (79) Range 319 to 643

Other ocular pathologies (eyes, %)		
None	40 (85.1%)	29 (55.7%)
Dry AMD	3 (6.4%)	8 (15.3%)
CRVO	—	1 (1.9%)
Glaucoma	1 (2.1%)	3 (5.8%)
Myopia	1 (2.1%)	2 (3.8%)
Corneal decompensation	1 (2.1%)	1 (1.9%)
Retinal tears	1 (2.1%)	3 (5.8%)
RD surgery	—	1 (1.9%)
Keratoconus	—	1 (1.9%)

ERM configuration (eyes, %)		
Preserved foveal pit	6 (12.8%)	3 (5.8%)
Absence of foveal pit	18 (38.3%)	7 (13.5%)
Prominent retinal thickening	20 (42.6%)	22 (42.3%)
Anteroposterior traction	3 (6.4%)	15 (28.8%)
Anteroposterior traction and retinal edema	—	5 (9.6%)

AMD, age-related macular degeneration; CRVO, central retinal vein occlusion; RD, retinal detachment.

**Table 2:** Status two years after diagnosis (follow-up group) or ERM surgery (vitrectomy group) and at the last follow-up visit. Values provided are mean (standard deviation) and range.

	Observation group	Vitrectomy group	*p* (Mann–Whitney)
*24 months*			
LogMAR visual acuity	0.14 (0.14) Range 0.50 to 0.0	0.16 (0.28) Range 1.30 to 0	0.528
Change in logMAR visual acuity from diagnosis	−0.07 (0.18) Range −0.50 to +0.40	−0.14 (0.29) Range −0.80 to +0.80	<0.001
Central macular thickness (*µ*m)	410 (49) Range 284 to 515	359 (59) Range 153 to 489	<0.001
			

*Last follow-up visit (months)*	43.2 (18.5) Range 24 to 105	30.3 (9.9) Range 6 to 48	0.001
LogMAR visual acuity	0.12 (0.15) Range 0.70 to 0.0	0.19 (0.28) Range 1.30 to 0	0.528
Change in logMAR visual acuity from diagnosis	−0.03 (0.13) Range −0.40 to +0.40	−0.13 (0.30) Range −0.80 to 0.80	0.002
Central macular thickness (*µ*m)	407 (49) Range 277 to 500	356 (61) Range 153 to 479	<0.001

## Data Availability

The data used to support the findings of this study are available from the corresponding author upon request.
